# Mixed-species groups and aggregations: shaping ecological and behavioural patterns and processes

**DOI:** 10.1098/rstb.2022.0093

**Published:** 2023-06-05

**Authors:** Nora V. Carlson, Todd M. Freeberg, Eben Goodale, Anne Heloise Theo

**Affiliations:** ^1^ Department of Biology, University of Victoria, Victoria, British Columbia, Canada V8W 2Y2; ^2^ Department of Psychology, University of Tennessee, Knoxville, TN 37996, USA; ^3^ Department of Health and Environmental Science, Xi'an Jiaotong-Liverpool University, Suzhou, Jiangsu 215123, People's Republic of China; ^4^ Centre for Ecological Sciences, Indian Institute of Science, Bangalore, Karnataka 560012, India

**Keywords:** sociality, species interactions, keystone species, mutualism, community assembly, facilitation

## Abstract

Mixed-species groups of birds, fishes and mammals have traditionally been described in taxa-specific journals. However, mixed-species systems are actually more widely found when one includes aggregative (non-moving) systems, such as those common in amphibians and invertebrates. The objective of this special issue is to dispel the idea that mixed-species phenomena are a ‘niche topic’ to ecology and instead explore how taking a mixed-species perspective can change our conception of important ecological patterns and processes. A mixed-species perspective starts by understanding the relative abundance and positioning of individuals of different species and their behavioural synchrony; it is enriched by understanding differences between species in their vulnerability/attractiveness to predators, their potential for competing with other group participants and their use as a source of public information. Contributions to the special issue show how the mixed-species perspective can change our ideas about invasion ecology, island biogeography, keystone species, mimicry, predator eavesdropping and more. Rather than seeking synthesis, the special issue celebrates the taxonomic and conceptual breadth of the field of mixed-species groups, with detailed descriptions of many distinctive systems.

This article is part of the theme issue ‘Mixed-species groups and aggregations: shaping ecological and behavioural patterns and processes’.

## 1. Diverse taxa but common questions

Mixed-species animal groups and aggregations include some of the most dramatic phenomena that humans can observe in the animal kingdom. These groups and aggregations include flocks of birds that can reach up to 60 species and 100 individuals [1], the iconic mixed herds on African plains [2], and groups of schooling fishes chased by larger predators (tuna and dolphins) and further attacked by seabirds from above [3]. At the same time, they also include groups and aggregations that are difficult for humans to encounter, like mixed groups of oceanic copepods [4], or ones that we may only appreciate aurally, such as chorusing groups of frogs [5] or orthoptera [6], or cannot perceive without specialized tools, like the larvae of insects in a cadaver [7]. However, despite their ubiquity, mixed-species groups are sometimes considered a ‘niche topic’ in ecology. This special issue is dedicated to dispelling this idea and displaying the diversity of taxa that are found in mixed-species contexts. Additionally, and just as importantly, we want to highlight the range of questions and approaches that have been applied to specific group types and encourage them to be explored across a wider variety of systems.

Mixed-species group phenomena have historically been described separately within taxonomic boundaries, with little cross-talk between these streams of research. Reports about moving groups have traditionally been in three clusters: birds [8], social mammals [9] and fishes [10]. A few reports have described ‘unlike fellows’ of mixed taxa, such as those formed by non-human primates and their heterospecific followers [11]. There have been a few attempts to compare and contrast across taxa [12–14] that have recently resulted in a book [15] and a cross-taxa review on moving groups [16]. Nevertheless, these syntheses are still incomplete because they have all focused on moving phenomena, and not on stationary aggregations that can occur either around resources, predators (such as in mobbing) or predator-free space, systems that are as common in invertebrates as vertebrates [17]. Because such aggregations are directly tied to the environment at one location, there are additional forces beyond species interactions that structure them. Nonetheless, aggregations and mixed-species groups share many similarities: in both cases animals are performing similar behaviours in close proximity, experiencing reduced predation (through risk dilution, ‘many eyes’ phenomena, predator assessment, active evasion or mobbing; [18]) and potentially increased competition owing to their grouping.

Our special issue is representative of the historical development of the field, as we include a large range of bird flock studies (10 that specifically work on or apply to moving groups of birds), two mammal studies, one fish study and two papers that aim to be applicable to multiple taxa. We also include aggregation systems: one each for amphibians, birds, insects, and a system of fishes and pelagic mammals. In bringing the studies on aggregations into the fold with moving systems, our hope is to show that a range of ‘mixed-species’ questions—shown perhaps best in the bird literature—can be applied to a much wider range of other taxa. Indeed, some of the issues of how groups of species in close proximity interact might be relevant to the burgeoning research field focused on plant communication and behaviour [19,20]. We would also like to note that another special issue coming out in *Philosophical Transactions of the Royal Society, B*, entitled ‘Collective behaviour through time’ (edited by Christos Ioannou and Kate Laskowski) will also cover some exciting mixed-species systems.

What then are the common themes across all this diversity? First, we are talking about groups of animals (and possibly microorganisms) in mobile life stages that are of the same trophic level. Basic ‘mixed-species’ questions that can be asked of these groups include:

(i) how many and which species are present in a group, how do they differ in their relative abundance, and how stable are these characteristics over time and space?
(ii) what is the spatial organization of individuals in these groups? How far are individuals typically apart from each other? Is the spacing between individuals different for conspecifics and heterospecifics?
(iii) if the whole system moves together, which species lead and which species follow? Is this consistent across different contexts and different group compositions? If the whole system is stationary, which species initiate aggregation at a location and which arrive subsequently?
(iv) how synchronous or asynchronous is individual behaviour, for both conspecifics and heterospecifics? This could include movements, foraging activities or communicative signals;
(v) how does information flow through the group? Is it unidirectional or multidirectional? Is it mutualistic or does it typically include aspects of eavesdropping/parasitism?
(vi) how does predation influence these groups? Does predation pressure increase group formation? Do group participants share the same level of vulnerability to the same predators or does this vary across species? Are some species in these groups preferred by predators and therefore each species benefits differently in both degree and type of benefits they receive in the group?
(vii) how does competition influence group structure (composition and organization) and how does this relate to an individual's proximity to resources? How do dominance hierarchies influence group structure?
(viii) how does a specific species composition affect a group's functioning, and how can we effectively experimentally remove or add individuals of different species or simulate their absence/presence to investigate this question?
(ix) how does the environment (e.g. gradients and habitat structure) affect, or even dictate, local group structure?
(x) how does the grouping phenomenon itself affect the composition of the whole community, as well as how that community uses resources? and
(xi) how does the grouping phenomenon affect the participants' responses to human disturbance and how can this information be used to improve conservation approaches?

With the inclusion of aggregations, it is clear that mixed-species phenomena are ubiquitous. Further, the breadth of these kinds of questions, from those that focus on the behaviour of individuals, to those that probe questions of community assembly, suggest that mixed-species issues are not a niche, but rather a core topic of ecological study. Below we argue further that thinking about the differences among participants in mixed-species groups can fundamentally alter the way we think about ecological patterns and processes.

## 2. The organization of the special issue

In the last section of Goodale *et al*. [16] the authors assessed how mixed-species groups might influence the structure and organization of communities. In the first section of this special issue, we follow up on this idea, showing some examples where an examination of mixed-species groups brings a unique perspective on a well-known issue in ecology. For instance, Bangal & Sridhar [21] review the citation history of the ‘nuclear species’ concept in bird flock systems and find that early framings of the concept have influenced the development of the ideas as much as the data. Camacho-Cervantes *et al*. [22] examine the important theoretical and applied field of invasion ecology, finding evidence that grouping with native species is often an important stage in the invasion process of multiple taxa. Kimball *et al*. [23] explore how phenotypic convergence in mixed-species groups, including mimicry, could lead to divergence within populations of species that interact with different group types. Martínez *et al*. [24] revisit the classic theory of island biogeography, finding that interspecific sociality influences the colonization and extinction rates of birds on islands. Finally, Trillo *et al*. [25] study predator eavesdropping on mating choruses, modelling how differences among species in their attractiveness and vulnerability influence parasite attack and disease transmission.

The second section is behavioural in focus, with the unifying concept being the comparison between intraspecific and interspecific sociality. We start with several papers that chronicle in depth the proximity of species to each other and their behavioural synchrony in a fascinating set of different contexts: captive South American monkeys by Daoudi-Simison *et al*. [26], groups of actively migrating birds by Gayk & Mennill [27], and pelagic hunting groups of fishes and mammals by Hansen *et al*. [28]. This section also includes a series of studies that look at how decisions, contests and associations are influenced by differences among mixed-group participants. Coppinger *et al*. [29] provide a conceptual framework for understanding how dominance shapes mixed-species societies. Freeberg *et al*. [30] describe an experimental study designed to see how the composition and diversity of groups affects novel food-finding behaviour. Ferdinand *et al*. [31] use a large dataset of mixed-species parrot aggregations to understand what characteristics of existing groups influence flock-joining decisions. Finally, Krama *et al*. [32] use a mixed-species tit system they have observed for more than 20 years to understand detailed relationships among individuals, in this case showing that juvenile birds stop alarm calling at the end of winter, potentially leading to more breeding opportunities in spring.

The third section asks what effects mixed-species systems have at the community level. Here the unifying concept is exploring how behavioural interactions between species influence species distributions and community composition across abiotic or biotic gradients. Charabidze & Aubernon [33] chronicle how maggot species are displaced from their optimal temperatures when aggregating with other species, and how the order of colonization influences the outcome. Fanjul *et al*. [34] examine the importance and influence of vegetation structure on mixed-species flock metrics and composition; this study also acts as an important intermediary site located between well-known Amazonian systems and much less diverse systems in Patagonia. Mangini *et al*. [35] review the mixed-species bird flock literature and attempt to classify the variation in group composition and the adaptive benefits that underlie groups, across all mixed-species groups in birds, proposing a common language for future studies. Montaño-Centellas *et al*. [36] examine the effect of environmental stress (elevation and latitude) on bird flocks networks of the Andes in order to test the hypothesis that increased environmental stress results in increased facilitative interactions. Munoz & Jankowski [37] investigate how mixed-species flocks and the overall bird community influence each other across a large mega-diverse elevational gradient. Saltz *et al*. [38] use an immense dataset of camera trap pictures to determine the conditions that promote mixed-species ungulate groups over single-species ones. Finally, Theo & Shanker [39] describe how different types of coral fish shoals responded to a mass bleaching event and subsequent reef recovery.

## 3. Research networks about animal networks

One final storyline for this special issue is also social in nature, but focused on human sociality, for this special issue developed out of an online symposium in June 2021. For Kathryn Sieving and Eben Goodale, this was the third such mixed-species group symposium in a decade, after a 2011 meeting sponsored by the Field Ornithology Group of Sri Lanka, and one in 2015 at Ecosummit in Montpellier, France, that was the first to attempt to mix researchers who work on different animal taxa. The topic of bird sociality and interspecific information flow has also been the focus of symposia before ([40,41,42]; as well as ‘Social Dynamics in Interspecific Interactions’, organized by Daizaburo Shizuka and Allison Johnson at the American Ornithological Society conference in 2019). We hope to make such mixed-species group symposia occur repeatedly in the future with online accessibility that allows students and researchers to participate across the world without large expenses. The geographical diversity of this special issue is clearly one of its strengths, as geographical diversity is important to truly understand any globally distributed biological phenomenon. Our theme issue is also enhanced by diversity in gender and the career stage of the scientists represented here, bringing different perspectives.

This special issue can be seen as building on the results of the 2015 Ecosummit, which developed into a synthesis article [16]. In that article, bird, fish and mammal researchers tried to develop a framework that could be used to categorize any kind of mixed-species group system, and specifically one that classified groups based on the similarities of their participants and the strength of leadership. At the end of that article, the contributors chronicled ways in which mixed-species phenomena can influence our perspective on major ecological issues. In this special issue, several of these ideas are given their own in-depth treatment. Putting together a special issue after the 2021 symposium has led us through a different, and equally interesting process, however, as each contribution is so different. Rather than synthesis and unification, we aim here for diversity in taxonomy, habitat, and in organization focus (behaviour to communities)—a special issue that celebrates the breadth of this field and is able to go into detailed description of many distinctive systems.

What directions can we go in the future, as a research network focusing on animal networks? Mixed-species group systems are very relevant to the emerging topics of the day, including biodiversity crisis, climate change and disease transmission. One major direction would be to use an international group of people who have contributed to this and earlier symposia, or who are inspired by this special issue, to plan joint data collection efforts, and ones that could sample across taxa and regions. For example, studies could address questions like how similarities between species affect the strength of associations among members, or how the diversity of the overall community in any one area is related to the diversity of the mixed-group systems there. Ultimately, by collecting similar data from a variety of organisms in a range of habitats and biomes, we can finally begin to comprehensively address the host of questions about mixed-group phenomena and the inherently multi-species communities that most animals live in.

In closing, we want to dedicate this special issue to the memory of Anne Heloise Theo (1985–2023). Anne passed away unexpectedly towards the end of the preparation of this special issue. She was an active member of our team, handling five of these manuscripts, and we are deeply grateful for her contribution, as well as saddened by losing her. Her passion for mixed-species systems and her beloved fish shoals shines forth in these pages.

## Data Availability

This article has no additional data.
